# Active Retrograde Extra Backup with a Mother-and-Child Catheter to Facilitate Retrograde Microcatheter Collateral Channel Tracking in Recanalization of Coronary Chronic Total Occlusion

**DOI:** 10.1155/2020/4245191

**Published:** 2020-08-31

**Authors:** Yong Wang, Xiao-Jiao Zhang, Hong-Wei Zhao, Cheng-Fu Wang, De-Feng Luo, Qing-Kun Meng, Yu Zhu, Jie Tao, Bao-Jun Chen, Yi Li, Ai-Jie Hou, Bo Luan

**Affiliations:** Department of Cardiology, The People's Hospital of China Medical University, The People's Hospital of Liaoning Province, Shenyang, China

## Abstract

**Objective:**

To explore the feasibility and safety of the active retrograde backup (ARB) for treatment of chronic total occlusion (CTO) during retrograde percutaneous coronary intervention (PCI).

**Background:**

Guiding support plays an important role in guidewire and microcatheter coronary channel (CC) tracking in retrograde PCI therapy for patients with CTO. However, the feasibility and safety of retrograde active use of a mother-and-child catheter are still unclear. *Patients and Methods.* A total of 271 consecutive patients with CTO who underwent retrograde PCI between January 2015 and January 2020 were retrospectively analyzed. Clinical data of two groups were compared to evaluate the feasibility and safety of ARB.

**Results:**

Of the 271 patients, 69.0% (187/271) underwent therapy through the septal branch, 31.0% (84/271) through the epicardial collateral channel, and 47.6% (129/271) through active retrograde extra backup with a mother-and-child catheter to facilitate retrograde microcatheter collateral CC tracking. The time of wire CC tracking was shorter in the ARB group than that in the non-ARB group (25.4 ± 8.5 vs 26.4 ± 9.7, *p*=0.348), but there was no significant difference. The duration of the retrograde microcatheter tracking (10.2 ± 3.8 vs 15.5 ± 6.8, *p*=0.012) and the retrograde approach (62.8 ± 20.3 vs 70.4 ± 24.3, *p*=0.026) in the ARB group was significantly shorter than that in the non-ARB group. The radiation dose (223.6 ± 112.7 vs. 295.2 ± 129.3, *p*=0.028), fluoroscopy time (50.6 ± 21.3 vs 62.3 ± 32.1, *p*=0.030), and contrast volume (301.8 ± 146.7 vs 352.2 ± 179.5, *p*=0.032) in the ARB group were significantly lower than that in the non-ARB group. There were no life-threatening procedural complications in either group. Complications unrelated to ARB included two cases of donor-vessel dissection, one case of CC perforation, and two cases of target-vessel perforation. There was no statistically significant difference in major adverse cardiac and cerebrovascular events between the groups during hospitalization (*p* > 0.05).

**Conclusion:**

ARB is feasible, safe, and conducive to guidewire and microcatheter CC tracking in the recanalization of coronary CTO. It improves procedural efficiency and is worthy of further promotion.

## 1. Introduction

Chronic total occlusion (CTO) lesions occur in 16–18% of patients with coronary artery disease undergoing coronary angiography [1, 2]. Before the introduction of the retrograde approach, the success rate of revascularization of CTO lesions was approximately 70% [3]. However, with advances in devices and new techniques, the most important being the introduction of the retrograde approach, the probability of success is above 90% at present. Nevertheless, retrograde interventional therapy for CTO-percutaneous coronary intervention (PCI) still faces great challenges. The primary retrograde approach may be considered as an alternative in patients who failed in antegrade CTO-PCI or those who have good collateral circulation.

The core of the retrograde interventional therapy is microcatheter tracking after successful guidewire tracking. In some cases, even if the guidewire passes through, retrograde microcatheter coronary channel (CC) tracking remains a challenge. Clinically, we found that the failure of microcatheter tracking in some patients was related to the poor supporting ability of retrograde guiding, and in cases where the retrograde guidewire has passed through the collateral circulation, replacing guiding is not the first choice. Retrograde passive delivery of a mother-and-child catheter to enhance support is a good approach, but it can lead to longer procedural time and increased X-ray exposure. Therefore, completing microcatheter CC tracking quickly and efficiently is important. In this study, we evaluated the feasibility and safety of Guidezilla (a mother-and-child catheter)-based active retrograde extra backup—termed active retrograde backup (ARB)—to facilitate retrograde microcatheter collateral channel tracking in the recanalization of CTO lesions.

## 2. Materials and Methods

### 2.1. Study Population

From January 2017 to January 2020, 271 consecutive patients with CTO who underwent retrograde interventional therapy successfully at the People's Hospital of Liaoning Province were enrolled in this study. The patients either had failed antegrade interventional therapy or had good retrograde collateral circulation with high chances of success with the primary retrograde interventional therapy. They were randomly divided into the ARB and non-ARB groups. All procedures were performed by Luan Bo (the corresponding author). The patients were administered loading doses of aspirin 300 mg and clopidogrel 300 mg (or ticagrelor 180 mg) before the procedure, and thereafter, aspirin 100 mg once daily and clopidogrel 75 mg once daily (or ticagrelor 90 mg twice daily) as dual antiplatelet treatment regimen. The routine access for the procedure was the radial artery, and the physician determined the access as needed. During the procedure, a standard dose of unfractionated heparin (100 IU/kg) was administered for anticoagulant therapy, and 2000 IU was added every hour. The use of glycoprotein IIb/IIIa inhibitor (GPI) was left to the physician's discretion. The study was approved by the Institutional Review Board of the People's Hospital of Liaoning Province. This study complied with the Declaration of Helsinki, and all patients signed informed consent before participation.

### 2.2. Definitions

CTO was defined as thrombolysis in myocardial infarction (TIMI) grade-0 flow for a duration of more than 3 months, angiographically documented or clinically defined [4]. CC grade was defined by Werner as follows: CC0, no continuous connection between donor and recipient vessel; CC1, continuous thread-like connection; and CC2, continuous, small side branch-like connection [5]. CC tortuosity was defined as the presence of more than two successive curves (within 2 mm) bent by >180° within a segment and less than three times the diameter of the collateral channel [6]. Adverse channel entry and exit angles were defined as a cut-off angle <90° [6]. For evaluating the antegrade PCI difficulty, the J-CTO score was used [7].

Retrograde guidewire tracking success was defined as the guidewire reaching the distal end of the occluded segment through collateral circulation without causing serious complications. Procedural success was defined as residual stenosis <30% and TIMI grade 3 for distal blood flow after stenting, without serious complications. Severe complications were defined as coronary artery perforation, dissection, hematoma, cardiac arrest, and pericardial tamponade, among others, requiring pericardiocentesis. Retrograde guidewire tracking time was defined as the time in which the guidewire entered from the retrograde CC ostial to the distal end of the occluded segment. Retrograde microcatheter tracking time was defined as the time from entry of the microcatheter at the CC ostial until it reaches the distal end of the occluded segment. Retrograde time was defined as the time from entry of the retrograde guidewire at the CC ostial until it reaches the antegrade guidewire, while passing through the occluded segment. All imaging data were recorded in detail and evaluated by two senior cardiologists. When their opinions differed, a third expert's opinion was sought.

### 2.3. Interventional Procedures

Procedural access was determined by the operator. Bilateral radiography was carried out for all patients to comprehensively evaluate the CTO lesions. The selection of collateral circulation, as well as the use and replacement of guidewires, was decided by the operator. First, a floppy wire carrying a 150 cm microcatheter was used to enter the CC ostial. When the guidewire reached the distal end of the occluded segment through the CC, the microcatheter was advanced to the distal end of the occluded segment, and then, the selection of the retrograde guidewire was at the operator's decision. For short lesions, retrograde wire crossing could be attempted. For long lesions, reverse controlled antegrade or retrograde subintimal tracking (CART) could be adopted.

### 2.4. Statistical Analysis

Statistical Package for the Social Sciences (SPSS) for Windows (version 20; IBM SPSS Inc., Chicago, IL, USA) was used for the statistical analysis. All normally distributed continuous data are expressed as mean ± SD, and those not normally distributed are expressed as median (range). Categorical variables are presented as numbers and percentages. To compare continuous variables, Student's *t*-test or Mann–Whitney *U* test was used. To compare categorical variables, the chi-square test and Fisher exact test were used in the case of sparse data. All tests were two-sided, and *p* values of <0.05 were considered significant.

## 3. Results

### 3.1. Baseline Characteristics

A total of 271 patients with CTO who underwent retrograde interventional therapy were included in this study. There was no significant difference in age, sex, body mass index, diabetes mellitus, hypertension, dyslipidemia, current smoker status, previous myocardial infarction, previous PCI, previous CABG, and LVEF (*p* > 0.05) (Table 1).

### 3.2. Angiographic and Procedural Characteristics

The femoral/radial approach was the most used access, accounting for 68.6% (186/271) of total cases. The right coronary artery was the most commonly involved in CTO lesions, accounting for 64.2% (171/271) of total cases. Angiography results showed that there was no significant difference in the procedural access, CTO vessels, in-stent CTO ratio, lesion characteristics (blunt stump at proximal cap, calcification, occlusion length >20 mm, side branch at landing zone, and J-CTO score), and CC characteristics (CC tortuosity and Werner score 0-1) (*p* > 0.05) (Table 2). Primary retrograde approach accounted for 73.8% (200/271) of cases, 69.0% (187/271) using the septal branch, and 31.0% (84/271) epicardial collateral branch. There was no significant difference between the two groups in terms of the adoption of the septal branch, epicardial collateral branch, or primary retrograde approach (*p* > 0.05) (Table 3). Although the time of wire CC tracking in the ARB group was shorter than in the non-ARB group (25.4 ± 8.5 vs. 26.4 ± 9.7, *p*=0.348), there was no significant difference. The time of the retrograde microcatheter tracking (10.2 ± 3.8 vs. 15.5 ± 6.8, *p*=0.012) and time of the retrograde approach (62.8 ± 20.3 vs. 70.4 ± 24.3, *p*=0.026) in the ARB group were significantly shorter than those of the non-ARB group. The radiation dose (223.6 ± 112.7 vs. 295.2 ± 129.3, *p*=0.028) and fluoroscopy time (50.6 ± 21.3 vs. 62.3 ± 32.1, *p*=0.030) in the ARB group were significantly lower than those of the non-ARB group. The contrast volume (301.8 ± 146.7 vs. 352.2 ± 179.5, *p*=0.032) in the ARB group was significantly lower than that in the non-ARB group. There was no significant difference between the two groups for the antegrade Guidezilla usage or reverse CART (*p* > 0.05) (Table 3).

There was no significant difference in procedural complications or in-hospital major adverse cardiac and cerebral events (MACCE). In the ARB group, no donor-vessel dissection occurred. In the non-ARB group, the incidence of donor-vessel dissection was 1.4% (2/142), and all cases were coronary artery ostial injury caused by guiding; these patients underwent stent implantation. No patient in the ARB group experienced CC perforation, while the incidence of CC perforation in the non-ARB group was 0.7% (1/142). The incidence of target-vessel perforation in the ARB group was 0.8% (1/129), while that of the non-ARB group was 0.7% (1/142). No cardiac tamponade was documented, and no death occurred in either group. The incidence of acute stent thrombosis in the ARB group was 0.8% (1/129). Target-vessel revascularization (TVR) was 0.8% (1/129) in the ARB group and 0.7 (1/142) in the non-ARB group (Table 4).

## 4. Discussion

This is the first study to confirm that the ARB technique can significantly improve the efficiency of retrograde PCI for patients with CTO. Its advantages include a shorter procedural time and a lower X-ray and contrast agent exposure without increasing procedure-related complications.

Septal branch or epicardial collateral circulation could be used in retrograde CTO-PCI. Several studies have confirmed that septal collateral circulation is safer than epicardial collateral circulation although the proportion of retrograde interventional therapy via epicardial collateral circulation has gradually increased in recent years. However, studies have confirmed that retrograde interventional therapy via epicardial collateral circulation has the same effectiveness and safety [8]. Guidewire tracking in a CC is a key step during retrograde CTO-PCI [7]. Previous studies have confirmed that vessel size, severe collateral tortuosity, side branch at CC tortuosity, inadequate CC exit, and Werner CC score were predictors of retrograde wire CC tracking failure [8–10]. Some studies have confirmed that the incidence of initial microcatheter CC tracking success was 77.5% in 280 procedures and that retrograde guiding support is an important factor for the success of microcatheter tracking [11].

In CTO-PCI, the guiding catheter must provide strong support for the passing of guidewire or for the subsequent advancement of microcatheter, followed by the balloon and stent. Therefore, the selection of a guiding catheter with a large lumen, strong support, and good coaxiality is a prerequisite for a successful CTO-PCI. In general, EBU, XB, and AL series of guiding catheters are used for CTO lesions in the left anterior descending branch, while AL series of guiding catheters are used for CTO lesions in the left circumflex coronary artery. For all CTO lesions in the right coronary artery, AL series of guiding catheters are considered. In contrast, JL and JR series of guiding catheters are rarely used for CTO lesions. However, rarely does an appropriate guiding catheter combine strong support and good coaxiality together. There are also other equipment and techniques to increase catheter support, such as the mother-and-child catheter, OTW catheter, deep catheter insertion, microcatheter, balloon anchoring, double guidewires, and multiple guidewires. However, problems can arise, such as intertwining of double and multiple guidewires, damage of branch vessels, perforation and dissection of the coronary arteries during balloon anchoring, and coronary artery ostial damage due to deep catheter insertion.

Guidezilla extension catheter provides stronger guiding support to facilitate the delivery of devices. It also increases coaxiality in order to (a) prevent the catheter from slipping accidentally, (b) overcome the different axes caused by arterial vascular abnormalities, and (c) prevent the catheter from exiting during the procedure.

At the same time, Guidezilla extension catheter prevents coronary artery ostial damage caused by strong guiding catheters and allows the use of guiding catheters with strong support and good coaxiality in CTO-PCI. However, guiding catheters with both qualities will increase the probability of coronary ostial damage, and deep insertion of guiding catheters carries a higher risk of coronary ostial and sinus damage. Guidezilla extension catheter together with an ordinary guiding catheter may not only prevent coronary artery ostial damage caused by a strong guiding catheter, particularly in patients with coronary artery ostial lesions, but could also improve the support required for CTO-PCI, reduce procedural complications, and increase the safety of the procedure. In this study, two cases of donor-vessel dissection occurred in the non-ARB group, both of which were due to coronary artery ostial dissection arising from the deep insertion of the catheter and were treated by stent implantation. The proportion of antegrade use of Guidezilla was similar between the two groups of patients. CTO lesions are often accompanied by tortuous, calcified, and diffuse lesions, as well as distal lesions [12], which increase the difficulty in guidewire tracking, as well as subsequent balloon and stent passing. The use of Guidezilla extension catheter enhances guiding support, improves coaxiality, and facilitates the entry of devices. In contrast, the use of antegrade Guidezilla is convenient for the retrograde guidewire to enter the antegrade guiding catheter, which is known as the active greeting technique (AGT) [13]. This study confirmed that the ARB technique can significantly reduce the procedural time and reduce X-ray exposure and contrast agent dosage, without increasing procedure-related complications. Combining the ARB technique with AGT can significantly improve the efficiency of retrograde interventional therapy for CTO lesions and is, thus, worthy of further promotion.

A previous meta-analysis showed that retrograde PCI for CTO lesions had a 6.9% incidence of target-vessel perforation and a 1.4% incidence of pericardial tamponade [14]. However, with advances in technology and devices, complications have steadily decreased in recent years, and the success rate has also increased significantly. This study included CTO patients who underwent successful retrograde intervention, with 0.74% (2/271) donor-vessel dissection and 0.74% (2/271) target-vessel perforation and without an occurrence of pericardial tamponade. Most procedures were performed by reverse CART mainly because the patients had higher J-CTO scores, and some of them had failed with previous antegrade interventions.

Regarding the timing of retrograde Guidezilla, we suggest the active use of retrograde Guidezilla, or ARB technique, to further improve the procedural efficiency. In order to obtain better supporting force and coaxiality, the head of Guidezilla entering the coronary artery should be sufficiently long although this may increase the risk of coronary artery injury. In particular, for patients with unstable plaques or completing balloon dilatation, we recommend that the head of Guidezilla entering the coronary artery should not exceed 15 cm. Before advancing Guidezilla, balloon-anchoring technology is recommended, and it is important to apply small-pressure expansion to avoid coronary artery injury. PCI is recommended when the donor vessel has severe stenosis or unstable lesions before interventional treatment of CTO lesions.

## 5. Limitations

The patients included in this study were from a single center and treated by the same operator; moreover, the patients were highly selected and the sample size was relatively small, and inevitably, there are some biases. The study patients were recruited from January 2017 to January 2020; the success rate of the procedure has been increasing annually with an increase in the number of procedure and progress in instruments, which resulted in different success rates between patients treated early and those treated recently, and this could have affected the study's results. The J-CTO scores of the subjects were relatively high and were not applicable to all study populations. In the future, data from multicenter, large sample size studies conducted by highly experienced centers and operators are needed to verify our conclusions.

## 6. Conclusions

ARB is feasible, safe, and conducive to guidewire and microcatheter CC tracking in the recanalization of coronary CTO. It improves procedural efficiency and is worthy of further promotion.

## Figures and Tables

**Table 1:** Clinical characteristics of study population.

Variables	ARB group (*n* = 129)	Non-ARB group (*n* = 142)	*p* value
Age (years)	65.8 ± 8.7	66.2 ± 9.1	0.427
Gender (female), *n* (%)	52 (40.3%)	55 (38.7%)	0.805
Body mass index (kg/m^2^)	22.7 ± 2.9	23.1 ± 3.5	0.394
Diabetes mellitus, *n* (%)	42 (32.6%)	45 (31.7%)	0.897
Hypertension, *n* (%)	39 (30.2%)	48 (33.8%)	0.603
Dyslipidaemia on admission, *n* (%)	38 (29.5%)	40 (28.2%)	0.893
Current smoker, *n* (%)	61 (47.3%)	66 (46.5%)	0.904
Previous MI, *n* (%)	35 (27.1%)	40 (28.2%)	0.892
Previous PCI, *n* (%)	96 (74.4%)	101 (71.1%)	0.586
Previous CABG, *n* (%)	9 (7.0%)	12 (8.5%)	0.821
LVEF (%)	42.1 ± 7.2	41.7 ± 7.5	0.717

MI, myocardial infarction; PCI, percutaneous coronary intervention; CABG, coronary artery bypass grafting; LVEF, left ventricular ejection fraction.

**Table 2:** Angiographic characteristics of the study population.

Variables	ARB group (*n* = 129)	Non-ARB group (*n* = 142)	*p* value
Access site			
Radial only	13 (10.1%)	18 (12.7%)	0.569
Femoral + radial	90 (69.8%)	96 (67.6%)	0.793
Femoral only	26 (20.2%)	30 (21.1%)	0.881

CTO vessel			
LAD-CTO, *n* (%)	37 (28.7%)	38 (26.8%)	0.786
LCX-CTO, *n* (%)	10 (7.8%)	14 (9.9%)	0.671
RCA-CTO, *n* (%)	82 (63.6%)	92 (64.8%)	0.899

Lesion characteristics			
In-stent CTO, *n* (%)	13 (9.4%)	16 (11.3%)	0.696
Blunt stump at proximal cap	58 (45.0%)	65 (45.3%)	0.903
Calcification	52 (40.3%)	55 (38.7%)	0.805
CC tortuosity, *n* (%)	48 (37.2%)	52 (36.6%)	1
Occlusion length >20 mm	102 (79.1%)	103 (72.5%)	0.257
Side branch at landing zone	58 (45.0%)	60 (42.3%)	0.713
J-CTO score	3.0 ± 1.1	3.1 ± 1.2	0.428
Werner score 0	9 (7.0%)	10 (7.0%)	1
Werner score 1	57 (44.2%)	70 (49.3%)	0.465
Werner score 2	63 (48.8%)	62 (43.7%)	0.464

CTO, chronic total occlusion; LAD, left anterior descending artery; LCX, left circumflex artery; RCA, right coronary artery; CC, collateral channel.

**Table 3:** Procedural characteristics of the study population.

Variables	ARB group (*n* = 129)	Non-ARB group (*n* = 142)	*p* value
Septal CC, *n* (%)	92 (71.3%)	95 (66.9%)	0.511
Epicardial CC, *n* (%)	37 (28.7%)	47 (33.1%)	0.511
Primary retrograde approach, *n* (%)	99 (76.7%)	101 (71.1%)	0.334
After failed antegrade approach, *n* (%)	30 (23.3%)	41 (28.9%)	0.334
Time of wire CC tracking (min)	25.4 ± 8.5	26.4 ± 9.7	0.348
Time of retrograde microcatheter tracking (min)	10.2 ± 3.8	15.5 ± 6.8	**0.012**
Time of retrograde approach (min)	62.8 ± 20.3	70.4 ± 24.3	**0.026**
Radiation dose (Gy. cm^2^)	223.6 ± 112.7	295.2 ± 129.3	**0.028**
Fluoroscopy time (min)	50.6 ± 21.3	62.3 ± 32.1	**0.030**
Antegrade Guidezilla usage, *n* (%)	40 (31.0%)	47 (33.1%)	0.795
Retrograde wire crossing, *n* (%)	14 (10.9%)	13 (9.2%)	0.688
Reverse CART, *n* (%)	115 (89.1%)	129 (90.8%)	0.688
Contrast volume (ml)	301.8 ± 146.7	352.2 ± 179.5	**0.032**

CC, collateral channel; CART, controlled antegrade and retrograde tracking. Bold values are statistically significant (*p* < 0.05).

**Table 4:** In-hospital outcomes.

Variables	ARB group (*n* = 129)	Non-ARB group (*n* = 142)	*p* value
Procedural complications			
Donor-vessel dissection, *n* (%)	0 (0%)	2 (1.4%)	0.499
CC perforation	0 (0%)	1 (0.7%)	1
Target-vessel perforation, *n* (%)	1 (0.8%)	1 (0.7%)	1
Cardiac tamponade, *n* (%)	0 (0%)	0 (0%)	—

In-hospital MACCE			
All-cause mortality, *n* (%)	0 (0%)	0 (0%)	—
Acute in-stent thrombosis, *n* (%)	1 (0.8%)	0 (0%)	0.476
TVR, *n* (%)	1 (0.8%)	1 (0.7%)	1

CC, collateral channel; MACCE, major adverse cardiac and cerebral event; TVR, target-vessel revascularization.

## Data Availability

The data supporting the results in the current study are available from the corresponding author on reasonable request.
